# Dental caries among children visiting a mobile dental clinic in South Central Kentucky: a pooled cross-sectional study

**DOI:** 10.1186/1472-6831-13-19

**Published:** 2013-05-02

**Authors:** Erika Dawkins, Akihiko Michimi, Gregory Ellis-Griffith, Tina Peterson, Daniel Carter, Gary English

**Affiliations:** 1 Department of Public Health, College of Health and Human Services, Western Kentucky University, 1906 College Heights Blvd, Bowling Green, KY 42101, USA; 2 Department of Social Work, College of Health and Human Services, Western Kentucky University, 1906 College Heights Blvd, Bowling Green, KY 42101, USA; 3 The Institute for Rural Health, College of Health and Human Services, Western Kentucky University, 1906 College Heights Blvd, Bowling Green, KY 42101, USA

**Keywords:** Dental health, Caries, Children, Mobile dental clinics, Kentucky

## Abstract

**Background:**

Dental caries is one of the most common chronic childhood diseases affecting a large portion of children in the United States. The prevalence of childhood dental caries in Kentucky is among the highest in the nation. The purposes of this study are to (1) compare sociodemographic differences between caries and no caries groups and (2) investigate factors associated with untreated dental caries among children who visited a mobile dental clinic in South Central Kentucky.

**Methods:**

Study subjects were children aged 6 to 15 years who participated in the school-based dental sealant program through the mobile dental clinic operated by the Institute for Rural Health at Western Kentucky University between September 2006 and May 2011 (n = 2,453). Descriptive statistics were calculated for sociodemographic factors (age, gender, race/ethnicity, insurance status, and urban versus rural residential location) and caries status. We used chi-square tests to compare sociodemographic differences of children stratified by caries and no caries status as well as three levels of caries severity. We developed a logistic regression model to investigate factors associated with untreated dental caries while controlling for sociodemographic characteristics.

**Results:**

The proportion of children having untreated dental caries was 49.7% and the mean number of untreated dental caries was 2.0. The proportion of untreated dental caries was higher in older children, children with no insurance and living in rural residential locations, and caries severity was also higher in these groups. Odds ratio indicated that older ages, not having private insurance (having only public, government-sponsored insurance or no insurance at all) and rural residential location were associated with having untreated dental caries after controlling for sociodemographic characteristics of children.

**Conclusions:**

Untreated dental caries was more likely to be present in older children living in rural areas without insurance. Health interventionists may use this information and target rural children without having proper insurance in order to reduce geographic disparities in untreated dental caries in South Central Kentucky.

## Background

Oral health plays an important role in maintaining a healthy human body. Good oral health enhances our ability to perform a variety of oral and ingestive functions, such as speaking, chewing, and swallowing; however, oral diseases, ranging from untreated dental caries (tooth decay) to oral cancer, cause pain and disability for millions of Americans each year [1]. In addition, poor oral health is associated with chronic diseases and ill health, such as cardiovascular disease and low-birth weight [2-4].

The Commonwealth of Kentucky exceeds the U.S. average for dental health problems as 13% of adults aged over 18 years are missing all of their teeth, compared to 6% nationally, placing Kentucky as the nation’s highest percentage of edentate (toothless) persons [5]. The prevalence of dental abnormalities, such as untreated caries, is also high among children in Kentucky [6,7]. Approximately 42.8% of Kentucky’s children before reaching the age of five have severe early childhood dental decay and 39.3% of these children have never visited a dentist [8]. Moreover, tooth decay is the single most common chronic childhood disease affecting 20% of preschoolers, 50% of second graders and nearly 75% of 15 year olds in Kentucky [9].

Rural residents in Kentucky are less likely to have dental insurance, compared to urban residents, and not having any form of dental insurance is associated with childhood dental caries [10,11]. Compared to rural areas, a greater proportion of residents living in urban areas have higher dental insurance coverage and dental care utilization rates but they do not necessarily have better dental health [11-13]. Various social and physical barriers to oral health care, such as no means of transportation to dental clinics and dentists not willing to accept Medicaid-insured children, are important issues related to poor dental health [14-16].

Governmental and non-governmental assistance programs, such as Medicaid, Kentucky Children’s Health Insurance Program (K-CHIP), and SMILE Kentucky, provide basic dental services for children from low income families. However, the utilization rate of dental services among Medicaid eligible children is low in Kentucky. Only 9.4% of Kentucky children eligible for Medicaid received early periodic screening, diagnosis, and follow-up treatment which was the lowest rate in the nation [9]. Research suggests that some children, despite having dental insurance, are not always receiving dental care because their parents are not able to take their children to dentists or not motivated enough to seek dental care for their children [17,18]. Untreated dental caries rates are high among children enrolled in public insurance, thus having government-assisted dental health insurance alone may not be fully effective in promoting better dental health [19].

Mobile dental clinics are another strategy to provide dental health care. Unlike stationary dental clinics, mobile clinics provide greater physical access to dental care for medically underserved populations in poor urban and remote rural communities, and many existing mobile dental clinics offer basic services at lower or no cost to the user [20,21]. School-based mobile dental programs are viable solutions to physical, financial, and structural barriers to dental care access for children [22,23]. Thus, children with all types of social, economic, and cultural backgrounds within predetermined geographic areas may participate in school-based dental care [24].

The Institute for Rural Health (IRH) at Western Kentucky University (WKU) is a university-based multidisciplinary organization that collaborates with several departments across the university. A dental sealant program is provided to school-aged children at no cost to their parents or guardians through the Mobile Dental Unit that travels to participating schools throughout South Central Kentucky. With federal funding and a budget from WKU’s College of Health and Human Services, the IRH has been providing services since 2001. Roughly 4,000 children have received preventive dental care services and dental examinations since the inception of the program.

This research reports on a pooled cross-sectional secondary data analysis which examines untreated dental caries among school-aged children (6 to 15 years old) who participated in the dental sealant program and received oral examinations via the Mobile Dental Unit operated by the IRH from September 2006 to May 2011. We investigated the sociodemographic differences of children by caries status as well as the degree of caries severity, and examined factors associated with untreated dental caries among children living in South Central Kentucky.

## Methods

Data were obtained from the Institute for Rural Health (IRH) at Western Kentucky University (WKU). We analyzed secondary data on children aged 6 to 15 years who participated in the dental sealant program provided by IRH clinicians through the Mobile Dental Unit (MDU). The staff consisted of a full-time dentist, and a full-time dental hygienist. Students enrolled in WKU’s Dental Hygiene program were supervised by the MDU clinicians and assisted with the dental procedures. The program was offered to primarily second and seventh grade students residing in South Central Kentucky because the first and second permanent molars appear around these ages [25]. The event locations were scheduled in advance, and appointments were made through the school where the service was provided.

**Figure 1:**
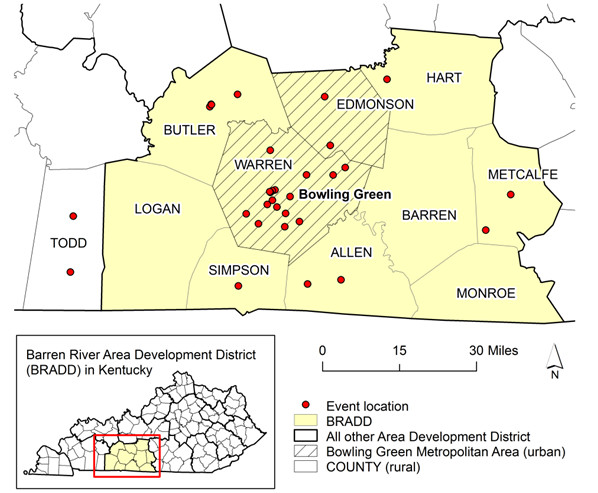
Mobile dental unit event locations in South Central Kentucky.

The event locations were mapped to show the general service area covered by the MDU. Events took place in 31 different locations during the study period (Figure 1). Nineteen locations (61.3%) were in Warren and Edmonson Counties which comprised the Bowling Green Metropolitan Statistical Area defined by the Office of Management and Budget (OMB) [26]. Metropolitan areas (urban) are characterized by a core urban county and/or adjacent counties containing a population of at least 50,000. Warren County contains the City of Bowling Green that has a population of more than 50,000 and Edmonson County has a strong social and economic connection to Warren County measured by commuting tie. In contrast, twelve locations (38.7%) were located outside the metropolitan area or so-called nonmetropolitan (rural) areas lacking major population centers. Thus, they are considered remote rural areas. The majority of events (93.5%) were located in the Barren River Area Development District (BRADD), a group of 10 counties encompassing South Central Kentucky. Two additional events were held outside this region.

The selection of schools was based on the availability of the IRH’s financial resources and coordination with local schools. The IRH initially started providing dental services to medically underserved children in selected counties within the BRADD and gradually expanded to other schools within the service area. All schools within the IRH’s primary service areas, i.e., the BRADD or South Central Kentucky, were eligible to participate in the dental program. All services were provided at no cost to the parents or guardians of the children. All children, regardless of insurance status, were eligible to receive the services. All children who participated in the program underwent oral exams prior to dental sealant application.

### Data collection procedure

All children were required to have a registration form completed by their parents or guardians prior to the service. The form included basic sociodemographic information, such as age, sex, race and ethnicity, new patient status, dental insurance coverage, and residential address. Parents or guardians were required to sign the general informed consent clause before the service was rendered to their children. The self-reported data on paper-based registration forms were transposed into a digital database by trained research assistants using Microsoft Access. Children who returned to the MDU more than once were excluded to avoid double counts.

Clinical data analyzed included the number of untreated dental caries. Dental caries is defined in a number of different ways in the literature [27]. For the purpose of this study, dental caries was defined as clinically detectable bacterial infections on external surface layer of teeth which causes demineralization and destruction of the hard tissues [28]. As part of the dental sealant program, oral examinations were performed by the IRH’s dentist and registered dental hygienist using a mirror, explorer, and air/water syringe in the fully equipped mobile dental unit. X-radiographs were not utilized. Dental hygiene students assisted the clinical staff during oral exams. Clinical data were entered into a separate table which was merged with the demographic data by patient ID. This research was approved by the Institutional Review Board at Western Kentucky University.

### Data analyses

The objective of this research was threefold. First, the descriptive statistics for the sociodemographic characteristics of children were calculated. Age was categorized into four groups (6–7, 8–9, 10–12, and 13–15 year olds). For race and ethnicity, non-white children were categorized into one group because of the small sample size. Insurance status was categorized into (1) private (dental insurance), (2) public (government-supported, e.g. Medicaid, K-CHIP), and (3) no insurance. Residential location was categorized into urban (the Bowling Green, KY metropolitan area) and rural (non-metropolitan areas) status according to the OMB definition. The number of dental caries was categorized into at least one tooth with untreated caries and no teeth with caries.

**Figure 2:**
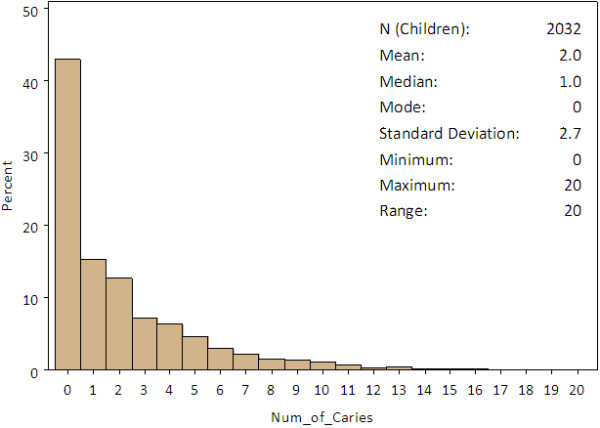
**Frequency distribution of the number of untreated dental caries and summary statistics.** Note: missing data and values greater than 20 were excluded.

Second, sociodemographic characteristics of children stratified by caries status were compared using chi-square tests. Caries status was categorized into dichotomy (caries versus no caries) and multiple caries categories. The rationale behind using multiple caries categories in analysis was to examine the degree or severity of dental health [29]. Different dental health indices and severity scores were used based on age of the subjects and research settings [30,31]. The frequency distributions of children with the number of untreated dental caries were plotted (Figure 2). Quantile classification method was used to categorize caries severity. In attempting to divide the distribution into roughly equal numbers of children, the following cutoffs of the number of teeth with caries were used: no caries (42.9%), 1 to 2 (28.0%) and ≥3 (29.1%). This classification method was used to ensure that each category would have a sufficiently large number of samples to conduct chi-square tests. The analysis of multiple caries categories was performed on variables found to be significant from the initial tests using dichotomous categories.

Third, factors associated with untreated dental caries were examined using multivariate logistic regression. Covariates included in the model were age, gender, race and ethnicity, insurance status, and residential location. Multicollinearity may apply to explanatory variables that are collinear [32]. Our logistic regression analysis identified that multicollinearity was not an issue with explanatory variables used in this research. Odds ratio (OR) estimates were summarized with 95% confidence intervals (CI). All analyses were carried out using SAS version 9.2.

## Results

Sociodemographic characteristics of the children are summarized in Table 1. A total of 2,453 children were seen at the MDU during the study period. The majority of children are between the ages of six and nine years. The sex ratio is roughly equal between male and female children. In our data, 82.2% were white, 5.8% were black, and 4.0% were Hispanic. The proportion of having private dental insurance is 44.7%, while that of having public insurance is 38.0%. The proportion of children who had no insurance is 10.7%. There are slightly more children living in rural areas (57.8%) than in urban areas (42.2%). The proportion of children who had at least one untreated dental caries is 49.7%.

**Table 1:** Descriptive statistics (n = 2453)

	**n**	**%**
Age, years		
6-7	893	36.4
8-9	1198	48.8
10-12	151	6.2
13-15	210	8.6
Gender		
Male	1199	48.9
Female	1243	50.7
Race and ethnicity		
White	2016	82.2
Black	142	5.8
Hispanic	98	4.0
Biracial	53	2.2
Asian/Pacific Islander	47	1.9
American Indian	2	0.1
No response	95	3.8
White and non-white		
White	2016	82.2
Non-white	342	14.0
Insurance coverage		
Private	1096	44.7
Public (government)	933	38.0
No insurance	262	10.7
No response	162	6.6
Residential location		
Urban	1035	42.2
Rural	1418	57.8
Caries status		
No caries	1205	50.3
Caries present	1221	49.7

Note: Sample characteristics for children aged 6 to 15 years who participated in the dental sealant program through the MDU operated by WKU’s IRH between September 2006 and May 2011. Missing data were excluded.

Sociodemographic differences of children stratified by caries status are summarized in Table 2. Age, insurance coverage, and residential location are statistically significant in caries status (*P* <0.001) while gender or race and ethnicity are not statistically significant in caries status. Among the no caries group, the proportion of the youngest group (6–7 year olds) is 39.2% and that of the oldest group (13–15 year olds) is 6.1%. Among the caries group, in contrast, the proportion of the youngest group is 33.7% and that of the oldest group is 11.1% showing the increasing trend of caries with increasing age. Among the no caries group, 54.3% of children had private dental insurance. Among the caries group, however, the majority had public insurance (44.6%) and the large proportion of children had no insurance (13.9%). Among the no caries group, 53.0% of children lived in rural areas while 62.9% lived in rural areas among the caries group.

**Table 2:** Sociodemographic differences of children by caries status

	**No caries**		**Caries**		***P *value**
	**n**	**%**	**n**	**%**	
Age, years (n = 2426)					
6–7	479	39.2	406	33.7	<0.001
8–9	595	48.7	590	49.0	
10–12	72	5.9	77	6.4	
13–15	75	6.1	132	11.0	
Gender (n = 2415)					
Male	578	47.4	608	50.8	0.093
Female	641	52.6	588	49.2	
Race and ethnicity (n = 2426)					
White	998	81.7	999	82.9	0.451
Non-white	223	18.3	206	17.1	
Insurance coverage (n = 2264)					
Private	622	54.3	464	41.5	<0.001
Public (government)	424	37.0	499	44.6	
No insurance	99	8.7	156	13.9	
Residential location (n = 2426)					
Urban	574	47.0	447	37.1	<0.001
Rural	647	53.0	758	62.9	

Note: The percent refers to column percentages. Due to rounding error, some totals will not equal 100%. Missing data were excluded.

Age, insurance coverage, and residential location are stratified by three caries categories (Table 3). Statistical significance remained after taking into account the severity of untreated dental caries. Among the youngest group, the proportion decreased as the severity of untreated dental caries increased (39.2%, 37.1%, and 31.4% for no caries, 1–2, and ≥3 groups, respectively). The reverse pattern is seen among the oldest group that the proportion increased as the severity of untreated dental caries increased (6.1%, 9.3%, and 11.5% for no caries, 1–2, and ≥3 groups, respectively). Children with private insurance had the decreasing trend in the severity of untreated dental caries with the largest proportion appearing in the no caries group (54.3%) and the smallest in the ≥3 group (34.9%). Children with public or no insurance, in contrast, had the increasing trend. Children living in rural areas experienced the increasing trend indicating that the severity of untreated dental caries is higher in rural areas than in urban areas.

**Table 3:** Sociodemographic characteristics of children by multiple caries categories

	**0 (No caries)**		**1 to 2**		**≥ 3**		
	**n**	**%**	**n**	**%**	**n**	**%**	***P *value**
Age, year							
6–7	479	39.2	211	37.1	185	31.4	0.001
8–9	595	48.7	269	47.3	302	50.9	
10–12	72	5.9	36	6.3	37	6.2	
13–15	75	6.1	53	9.3	68	11.5	
Insurance coverage							
Private	622	54.3	249	47.1	193	34.9	<0.001
Public (government)	424	37.0	229	43.3	257	46.7	
No insurance	99	8.7	51	9.6	101	18.4	
Residential location							
Urban	574	47.0	239	42.0	192	32.6	<0.001
Rural	647	53.0	330	58.0	400	67.4	

Note: The percent refers to column percentages. Due to rounding error, some totals will not equal 100%. Missing data were excluded.

The odds ratios (OR) for children’s caries status are summarized in Table 4. The oldest group is more likely to have untreated dental caries, compared to the youngest group (OR 1.53, 95% CI [1.08, 2.15]). There is no gender or racial differences in the likelihood of having untreated dental caries. Children who had private insurance are less likely to have untreated dental caries compared to children who had no insurance (OR 0.51, 95% CI [0.38, 0.69]). There is no difference in untreated dental caries between children who had public insurance and children who had no insurance. Rural children are more likely to have untreated dental caries compared to urban children (OR 1.28, 95% CI [1.06, 1.55]).

**Table 4:** Odds ratios for untreated dental caries (n = 2032)

	**OR, 95% CI**	***P*-value**
Age, years (ref. 6 to 7)		
8 to 9	1.00 [0.83, 1.21]	0.972
10 to 12	1.03 [0.70, 1.51]	0.888
13 to 15	1.53 [1.08, 2.15]	0.017
Gender (ref. female)		
Male	1.19 [0.99, 1.41]	0.053
Race/ethnicity (ref. White)		
Non-white	0.95 [0.73, 1.23]	0.687
Insurance (ref. no insurance)		
Private	0.51 [0.38, 0.69]	<0.001
Public (government)	0.83 [0.62, 1.12]	0.225
Residential location (ref. urban)		
Rural	1.28 [1.06, 1.55]	0.009

Note: Values in the parentheses are 95% confidence interval.

Missing data were excluded.

## Discussion

Our results were consistent in all stages of analysis, which indicated that age, insurance coverage, and residential location were important factors related to untreated dental caries in school-aged children in South Central Kentucky. Older children were more likely to have untreated caries than younger children. Health interventionists may use this information to prevent dental problems in older children. It is during childhood that habits begin to form and the earlier children start to learn good oral habits the greater the impact it will have on them later in life [33]. Messages about practicing good oral health habits can be reinforced during childhood development through providing dental education regularly. In addition, children in schools begin to make their own decisions and choices on what to eat [34,35]. School children are exposed to opportunities inside or outside school settings to purchase sugary beverages or snacks through vending machines [36]. Frequent consumption of sugary foods, along with poor dental hygiene may explain the higher prevalence of untreated dental caries among the older school children [37,38].

In Kentucky, Medicaid Dental Programs are offered to eligible children under the age of 21 and the coverage includes basic services, such as oral exams, x-rays, emergency visits, and fillings [39]. In this study, however, public or government-sponsored dental insurance plans seemed to have little impact on having less untreated dental caries. Children covered through private dental insurance had fewer dental caries compared to children with no insurance. This finding is consistent with other studies documenting that children with Medicaid and CHIP have higher prevalence of dental diseases compared to children with private insurance [40,41]. Children with Medicaid and public assistance insurance may have limited access to and utilization of dental care due to various social, economic, and cultural reasons that prevent them from seeking dental care [14]. Particularly, persistent poverty and low income may be directly or indirectly affecting children’s dental health [42].

This research showed significant urban–rural disparities in untreated dental caries, characterizing poor dental health among rural children. Contrary to our findings, national level studies suggest no differences in caries lesions and caries experiences between urban and rural children [43]. This may be related to a number of factors. First, different definitions and indices of caries and dental conditions may be used in various research settings [27,44]. Second, urban versus rural areas may be defined differently. A study from Louisville, Kentucky, for example, showed that children living in the Louisville metro area, defined by the city zip codes, were more likely to have untreated caries compared to children living outside the metro area [11]. Other research uses the metropolitan area-based definition which includes suburban or fringe counties of a metropolitan area as ‘urban’ [45]. Thus, different results may be obtained based on how urban and rural residential locations are defined and who resides in such locations. Lastly, it is important to note the possibility of data aggregation. Compared to national level studies, geographically disaggregated data may unmask subnational health disparities, thus, it is likely to see spatial variability of health events using data at the local level [46].

There are other factors that may be associated with higher prevalence of untreated dental caries in rural areas. Rural areas are prone to dentist shortage as the number of practicing dentists is projected to start declining in 2014 due to mass retirement of older dentists, while dental schools are producing fewer graduates, and some dentists are not willing to practice in rural areas [47]. Dental caries experience among children was lower in fluoridated communities than in non-fluoridated communities [48]. While some households with private well water supplies have excessive fluoride exposure, other households have lower fluoride levels, and many rural communities lack optimally fluoridated water supplies [49,50]. An additional factor to consider is the fact that residents of rural communities may have differing levels of knowledge, attitudes, and beliefs about oral health compared to urban residents which may impact caries outcomes [12,51].

There was no racial/ethnic difference in untreated dental caries in South Central Kentucky. The National Survey of Children’s Health, however, reports suboptimal dental health among the minority groups compared to non-Hispanic white children [52]. The majority of the non-white children in our study lived in urban areas (72%). Urban children, however, had less untreated dental caries even after controlling for racial/ethnic difference. The variation from the national trend in our study area should deserve greater attention and further research is needed to explain the absence of racial/ethnic disparities in untreated caries in South Central Kentucky.

### Limitations

This study was subject to several limitations. First, the sample size for the non-white groups was small, thus, we were not able to perform our analyses using more specific racial and ethnic groups than non-white. Recruiting children in non-white groups is an inherent problem in Kentucky because the percent of black and Hispanic residents, for example, is well below the national average [53]. In addition, our analyses did not include a more direct measure of socioeconomic status, such as family income which may impact children’s dental health. To compensate for this lack of data, we included insurance status and rural location as surrogates for family income.

A pooled cross-sectional analysis did not allow the same population to be observed over the study time periods. We were only able to assess sociodemographic differences of children by caries status. Following the same children from elementary schools to middle schools may provide more complete and accurate estimates of untreated dental caries and greater insights into the progression of dental health problems due to advanced age.

In this study, untreated dental caries served as the indicator of poor dental health. Other commonly used indices, such as the decayed-missing-filled teeth (DMFT) index, was not used because not all data were available. Using other indices may produce different results. In addition, we examined untreated dental caries by reporting odds ratios rather than other statistical methods such as prevalence ratios. It is preferable to estimate prevalence ratios instead of odds ratios in cross-sectional studies when disease is common [54]. Odds ratio, however, is a standard and practical method that fits the model with maximum likelihood estimates and requires fewer assumptions than prevalence ratio does [54,55]. Thus, our study is consistent with other epidemiological studies reporting odds ratios controlling for other factors.

Lastly, this research was conducted using a convenience sample of children whose parents had agreed to have their children participate in the dental sealant program provided by the mobile unit in schools. Children who participated in the program may have social, economic, and cultural traits that are different from ones who did not. The IRH targets medically underserved children, but all second and seventh grade children in participating schools were eligible to receive preventive dental care services regardless of their socioeconomic status. During the dental screening, however, some children were not cooperative and did not finish the complete procedures and/or examinations. In addition, we pooled samples from five academic years (September 2006 to May 2011) to increase sample sizes and statistical reliability. We excluded returned children to avoid double counts in all analyses.

## Conclusions

Older ages, public insurance or no insurance, and rural residential location were important factors associated with having untreated dental caries in school-aged children in South Central Kentucky. Gender and race/ethnicity, however, were not significant factors associated with untreated dental caries. This information may be useful in planning school-based dental programs and target children in rural areas without dental insurance in order to reduce dental health disparities.

## Competing interests

The authors declare that they have no competing interests.

## Authors’ contributions

ED and AM designed the study, performed data analyses, and drafted the original paper. DC supervised dental hygiene students, and examined and collected data during dental screenings. GE-G, TP, DC, and GE provided comments on the original draft and contributed to the development of the final draft. All authors read and approved the final manuscript.

## Pre-publication history

The pre-publication history for this paper can be accessed here:

http://www.biomedcentral.com/1472-6831/13/19/prepub

## References

[B1] Preventing cavities, gum disease, tooth loose, and oral cancers at a glance http://www.cdc.gov/chronicdisease/resources/publications/AAG/doh.htm

[B2] Boggess KA Beck JD Murtha AP Moss K Offenbacher S Maternal periodontal disease in early pregnancy and risk for a small-for-gestational-age infant Am J Obstet Gynecol 2006 194 5 1316 1322 10.1016/j.ajog.2005.11.059 16647916

[B3] Haraszthy VI Zambon JJ Trevisan M Zeid M Genco RJ Identification of periodontal pathogens in atheromatous plaques J Periodontol 2000 74 10 1554 1560 11063387 10.1902/jop.2000.71.10.1554

[B4] DeRossi SS Sollecito TP The oral-medical disease connection: pregnancy, cardiovascular disease, and diabetes Compend Contin Educ Dent 2012 33 6 406 414 22774329

[B5] Childress MT Smith-Mello M Foresight: Kentucky's oral health poses challenges Long Term Policy Research Center 2007 50 Frankfort, KY http://www.e-archives.ky.gov/pubs/LPRC/foresighno50.pdf

[B6] Haelthy Kentucky Smiles A lifetime of oral health Statewide oral health strategic plan: the commonwealth of Kentucky, 2006 2006 Frankfort, KY: Kentucky Department for Public Health

[B7] A Project of the Child and Adolescent Health Management Initiativehttp://www.childhealthdata.org/home

[B8] Kentucky Oral Health Summithttp://chfs.ky.gov/dph/mch/cfhi/kohs.htm

[B9] Henry RG Kentucky's Dental Access Summit 2001 Lexington, KY: Kentucky Dental Health Coalition

[B10] Lu N Samuels ME Kletke PR Whitler ET Rural–urban differences in health insurance coverage and patterns among working-age adults in Kentucky J Rural Health 2010 26 2 129 138 10.1111/j.1748-0361.2010.00274.x 20446999

[B11] Kandel EA Richards JM Binkley CJ Childhood caries in the state of Kentucky, USA: a cross-sectional study BMC Oral Health 2012 12 38 10.1186/1472-6831-12-38 PMC3532194 22950640

[B12] Heaton IJ Smith TA Raybould TP Factors influencing use of dental services in rural and urban communities: considerations for practitioners in underserved areas J Dent Educ 2004 68 10 1081 1089 15466058

[B13] Byck GR Walton SM Cooksey JA Access to dental care services for Medicaid children: variations by urban/rural categories in Illinois J Rural Health 2002 18 4 512 520 10.1111/j.1748-0361.2002.tb00918.x 12380894

[B14] Kelly SE Binkley CJ Neace WP Gale BS Barriers to care-seeking for children's oral health among low-income caregivers Am J Public Health 2005 95 8 1345 1351 10.2105/AJPH.2004.045286 16043666 PMC1449365

[B15] Davis EE Deinard AS Maiga EW Doctor, my tooth hurts: the costs of incomplete dental care in the emergency room J Public Health Dent 2010 70 3 205 210 10.1111/j.1752-7325.2010.00166.x 20337900

[B16] Mofidi M Rozier RG King RS Problems with access to dental care for Medicaid-insured children: what caregivers think Am J Public Health 2002 92 1 53 58 10.2105/AJPH.92.1.53 11772761 PMC1447388

[B17] Larsen CD Larsen MD Handwerker LB Kim MS Rosenthal M A comparison of urban school- and community-based dental clinics J Sch Health 2009 79 3 116 122 10.1111/j.1746-1561.2008.00395.x 19207517

[B18] Amin MS Utilization of dental services by children in low-income families in Alberta J Can Dent Assoc 2011 77 b57 21627867

[B19] Brickhouse TH Rozier RG Slade GD Effects of enrollment in Medicaid versus the State Children's health insurance program on kindergarten children's untreated dental caries Am J Public Health 2008 98 5 876 881 10.2105/AJPH.2007.111468 18382008 PMC2374820

[B20] Gardner T Gavaza P Meade P Adkins DM Delivering free healthcare to rural Central Appalachia population: the case of the Health Wagon Rural Remote Health 2012 12 2035 22452285

[B21] Mobile Health Maphttp://www.mobilehealthmap.org/

[B22] Skillman SM Doescher MP Mouradian WE Brunson DK The challenge to delivering oral health services in rural America J Public Health Dent 2010 70 Supple 1 S49 57 20806475 10.1111/j.1752-7325.2010.00178.x

[B23] Jackson DM Jahnke LR Kerber L Nyer G Siemens K Clark C Creating a successful school-based mobile dental program J Sch Health 2007 77 1 1 6 10.1111/j.1746-1561.2007.00155.x 17212753

[B24] Albert DA McManus JM Mitchell DA Models for delivering school-based dental care J Sch Health 2005 75 5 157 161 15989084

[B25] Ekstrand KR Christiansen J Christiansen ME Time and duration of eruption of first and second permanent molars: a longitudinal investigation Community Dent Oral Epidemiol 2003 31 5 344 350 10.1034/j.1600-0528.2003.00016.x 14667005

[B26] Update of statistical area definitions and guidance on their uses 2009 Washington, D.C.: Executive Office of the President, Office of Management and Budget http://www.whitehouse.gov/sites/default/files/omb/assets/bulletins/b10-02.pdf

[B27] Pitts NB Modern concepts of caries measurement J Dent Res 2004 83 Spec No C C43 47 15286121 10.1177/154405910408301s09

[B28] Kidd EAM Fejerskov O What constitutes dental caries? histopathology of carious enamel and dentin related to the action of cariogenic biofilms J Dent Res 2004 83 Spec Iss C C35 C38 15286119 10.1177/154405910408301s07

[B29] Ferreira SH Beria JU Kramer PF Feldens EG Feldens CA Dental caries in 0- to 5-year-old Brazilian children: prevalence, severity, and associated factors Int J Paediatr Dent 2007 17 4 289 296 10.1111/j.1365-263X.2007.00831.x 17559457

[B30] Gherunpong S Sheiham A Tsakos G A sociodental approach to assessing children's oral health needs: integrating an oral health-related quality of life (OHRQoL) measure into oral health service planning Bull Qorld Health Organ 2006 84 1 36 42 10.2471/blt.05.022517 PMC2626510 16501713

[B31] Peres MA de Oliveira Latorre Mdo R Sheiham A Peres KG Barros FC Hernandez PG Maas AM Romano AR Victora CG Social and biological early life influences on severity of dental caries in children aged 6 years Community Dent Oral Epidemiol 2005 33 1 53 63 10.1111/j.1600-0528.2004.00197.x 15642047

[B32] Allison PD Logistic Regression Using SAS: Theory and Applications 2012 2 Cary, NC: SAS Institute, Inc.

[B33] Bertness J Holt K Promoting Oral Health in Schools: A Resource Guide 2009 Washington, D.C: U.S. Department of health and Human Services, Health Resources and Services Administration

[B34] Warash BG Fitch C Bodnowich K Snack choices: helping young children make decisions J Fam Consum Sci 2003 95 2 60 64

[B35] Blinkhorn AS Roberts BP Duxbury JT The ability of young children to influence adults in the choice of sugary foods and drinks Health Educ J 2003 62 3 210 219 10.1177/001789690306200303

[B36] Price J Murnan J Bradena M Soft drink vending machines in schools: a clear and present danger Am J Health Behav edu 2006 37 5 306 314

[B37] Mobley C Marshall TA Milgrowm P Coldwell SE The contribution of dietary factors to dental caries and disparities in caries Acad Pediatr 2009 9 6 410 414 10.1016/j.acap.2009.09.008 19945075 PMC2862385

[B38] Harris R Nicoll AD Adair PM Pine CM Risk factors for dental caries in young children: a systematic review of the literature Community Dent Health 2001 21 1 Supple 71 85 15072476

[B39] Department for Medicaid Services Dental Services 2012 12 Frankfort, KY: Kentucky Cabinet for Health and Family Services http://www.chfs.ky.gov/dms

[B40] GAO Office USGA Oral Health: Efforts under way to improve children's access to dental services, but sustained attention needed to address ongoing concerns 2010 Washington, D.C: The Government Accountability Office

[B41] Edelstein BL Disparities in oral health and access to care: findings of national surveys Ambul Pediatr 2002 2 2 Suppl 141 147 11950385 10.1367/1539-4409(2002)002<0141:diohaa>2.0.co;2

[B42] Bagramian RA Garcia-Godoy F Volpe AR The global increase in dental caries. A pending public health crisis Am J Dent 2009 21 1 3 8 19281105

[B43] Vargas CM Ronzio CR Hayes KL Oral health status of children and adolescents by rural residence, United States J Rural Health 2003 19 3 260 268 10.1111/j.1748-0361.2003.tb00572.x 12839134

[B44] Marshall TA Levy SM Broffitt B Warren JJ Eichenberger-Gilmore JM Burns TL Stumbo PJ Dental caries and beverage consumption in young children Pediatrics 2003 112 e184 e191 10.1542/peds.112.3.e184 12949310

[B45] Eberhardt MS Pamuk ER The importance of place of residence: examining health in rural and nonrural areas Am J Public Health 2004 94 10 1682 1686 10.2105/AJPH.94.10.1682 15451731 PMC1448515

[B46] Johnson PJ Call KT Blewett LA The importance of geographic data aggregation in assessing disparities in American Indian prenatal care Am J Public Health 2010 100 1 122 128 10.2105/AJPH.2008.148908 19910356 PMC2791242

[B47] Collier R United States faces dentist shortage Can Med Assoc J 2009 181 11 E253 E254 10.1503/cmaj.109-3086 19858248 PMC2780510

[B48] Gillcrist JA Brumley DE Blackford JU Community fluoridation status and caries experience in children J Public Health Dent 2001 61 3 168 171 10.1111/j.1752-7325.2001.tb03385.x 11603320

[B49] Graves JM Daniell W James F Milgrom P Estimating fluoride exposure in rural communities: a case study in Western Washington Wash State J Public Health Prac 2009 2 2 22 31 PMC2898138 20617156

[B50] NRHA Meeting oral health care needs in rural America National Rural Health Association. vol April 2005 2005 Kansas City, MO: National Rural Health Association

[B51] Patrick DL Lee RS Nucci M Grembowski D Jolles CZ Milgrom P Reducing oral health disparities: a focus on social and cultural determinants BMC Oral Health 2006 6 Suppl 1 S4 10.1186/1472-6831-6-S1-S4 16934121 PMC2147600

[B52] Dietrich T Culler C Garcia RI Henshaw MM Racial and ethnic disparities in children's oral health: the National Survey of Children's Health J Am Dent Assoc 2008 139 11 1507 1517 18978389 10.14219/jada.archive.2008.0077

[B53] State and County Quick Factshttp://quickfacts.census.gov/qfd/states/21000.html

[B54] Petersen MR Deddens JA A comparison of two methods for estimating prevalence ratios BMC Med Res Methodol 2008 8 9 10.1186/1471-2288-8-9 PMC2292207 18307814

[B55] Pearce N Effect measures in prevalence studies Environ Health Perspect 2004 112 10 1047 1050 10.1289/ehp.6927 15238274 PMC1247374

